# Evaluating building performance in healthcare facilities using entropy and graph heuristic theories

**DOI:** 10.1038/s41598-022-13004-8

**Published:** 2022-05-28

**Authors:** Amr A. Hassanain, Mohamed A. A. Eldosoky, Ahmed M. Soliman

**Affiliations:** Biomedical Engineering Department, Faculty of Engineering, Helwan University, Cairo, 11795 Egypt

**Keywords:** Biotechnology, Environmental sciences, Environmental social sciences, Health care, Engineering, Mathematics and computing

## Abstract

Designing a healthcare facility is one of the most challenging tasks due to the complexity associated with these facilities. The primary goal of healthcare facilities is to provide high-quality care; consequently, the design of healthcare facilities and their environments directly affects the facility's productivity, the organization's economic performance, the experienced clinical outcomes in the hospital, as well as patient and staff satisfaction. The redesign of a healthcare facility is essential for ensuring a serene healing environment for the patients and thus influences their healing rates, reduces the amount of time spent in the facilities, and impacts their level of satisfaction with the care provided. The evaluation methodology is a step in the redesign process that measures the performance of healthcare buildings according to international standards. In this study, the collected standards were weighted using an entropy algorithm to evaluate different departments in various hospitals. In addition, the layout score was measured using the adjacent algorithm as one of the graph heuristic methods to determine whether the department or the whole hospital can be redesigned to meet international standards. According to the results of our methodologies being used in one selected hospital in Egypt, the average of the satisfied standards was 43%, standards that could be satisfied were 24%, not applicable standards were 34%, and the average layout score was 25.

## Introduction

A hospital is made up of various units that work together to provide exemplary patient care. Some of the units involved include surgical suits, emergency areas, diagnostic imaging departments, critical care units, newborn intensive care areas, and laboratories. The design of a health facility is guided by its primary functions, which include research, inpatient and outpatient, diagnosis, or administration purposes^1^. When redesigning a hospital, the designer must consider the current status, such as the facility location, whom the facility serves, and the level of the facility expected. As the first step in the redesign process, this article aims to redesign the hospital by evaluating and weighing various standards in building a healthcare facility that meets international set requirements. American Institute of Architecture (AIA) Guidelines for Design and Construction of Health Care Facilities^2^, American Society of Heating, Refrigerating, and Air-Conditioning Engineering (ASHRAE)^3^, ventilation standards for healthcare facilities, and Health Technical Memorandum (HTM) guidelines for design, installation, validation, and verification of medical gas pipeline systems^4^, Facility guideline institution (FGI) for Design and Construction of Health Care Facilities^5^, were used as standard references for the evaluation process.

The implemented decision matrices can PROVIDE a definite structure in which various options can be compared to make a better decision. A weighted decision matrix compares a group of choices to the criteria considered in the decision-making process. Therefore, many researchers have developed a variety of weighting methods to assist with Materials and Methods. The materials and methods section should include adequate details to allow all procedures to be repeated. It may be divided into headed subsections if several methods are described. In decision making, including the analytic hierarchy process, critic method, and entropy method^6^.

### Entropy method

Entropy is a method used to evaluate the weight of a given problem, in which the decision conditions for a set of applicant materials contain a particular volume of information. R. Clausius, a physicist, predicted the thermodynamic theory of entropy in 1865. It is a formal factor of the mater that denotes the state of thermodynamic systems^7^. In 1948, Shannon presented entropy into the data concept, which was used to determine the ambiguity of indications of information source knowns as follows: information entropy. The entropy method is primarily used in information theory to signify communication ambiguity, assess the capacity for separate assessment power to permit decision data, and determine the virtual weight. As shown in the judgment matrix, the entropy weight can be intended. "The smaller the entropy of evaluated information criterion, the greater the weight of the information criterion^8^, which is a solitary factual if the fundamental expectations that the entire foundations of information are dependable.

**Figure 1:**
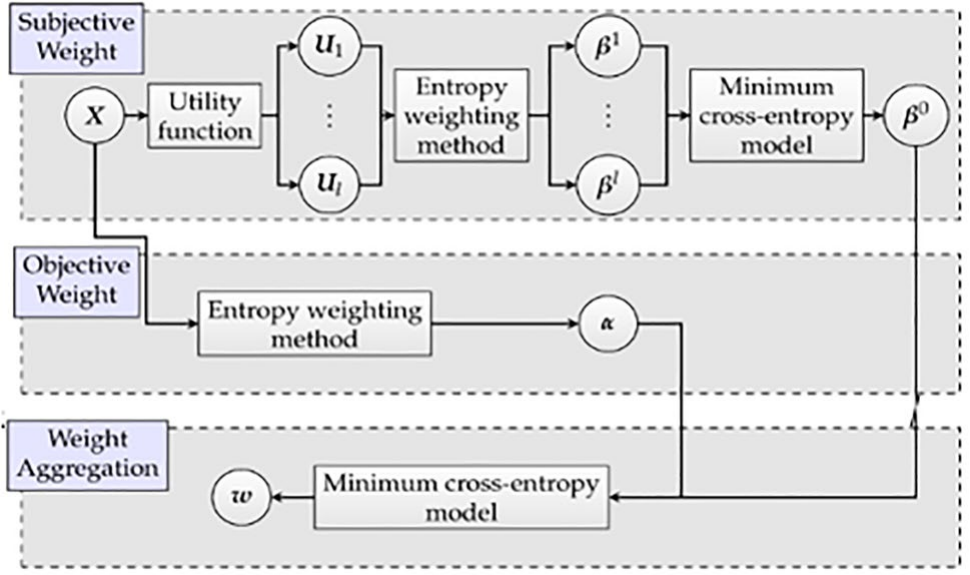
The method of determining and aggregating weight source.

The entropy method is significant for several reasons: it estimates the information of the sign in addition to the practice point of the variance of the sign to determine the actual information and sign weight kept in check in the identified data. The weighting of entropy specifies the virtual significance of the constant sign in the struggle, which is under the circumstances of a specified appraisal thing, as demonstrated in Fig. 1.

### Benefits of the entropy method

This method is well known for effectively determining the divergence of responses and contrast intensity and reckoning their weights appropriately^9^.

Furthermore, it recommends that the available information is adequate, and if not enough, additional information is needed^10^.

This approach allows for the quantitative evaluation of the success and cost responses.

The entropy weight method plan provides an additional difference in coefficient approval for answers. It is appropriate for an entropy plan to determine a significant disagreement between the decision-making answers^11^.

The strategy of this method aids in the calculation of the weight and is an immensely effective technique for evaluating indicators.

### Limitation of the entropy method

The entropy computed weights missing specialist decisions; therefore, it only considers entropy values^12,13^.

This method does not provide any involvement in the designer's first choice.

The efficiency of the entropy method in making decisions demonstrates that the preference for this method is problematic, as it does not consider rank perception.

The entropy method is used for weighting our standards collected after most of the methods are analyzed for weighting, and the lack of a standard can be measured in any department to apply the evaluation process. However, we encounter a significant issue with the layout facilities; hence, graph-theoretic-based heuristics are used to measure the layout score compared to the criteria that must be considered in the decision-making process. Therefore, many researchers have developed a variety of weighting methods to help in decision making, including the analytic hierarchy process, critic method, and entropy method.

Multi-criteria decision-making analysis can be described as a research approach that assists in creating a complex decision through explicit consideration in a transparent manner, which is essential because it makes it easy to clearly understand the question, thereby improving the efficiency and consistency of the decision-making process, as described by^14^.

**Figure 2:**
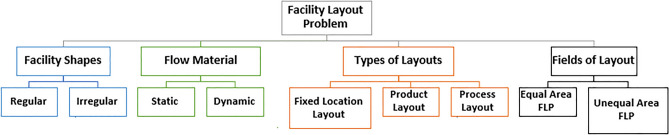
Facility layout problems.

**Figure 3:**
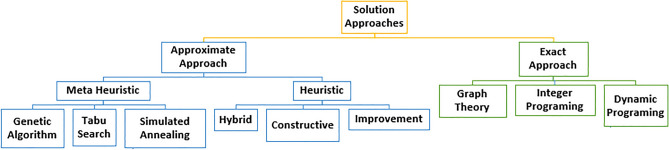
Facility layout problems.

The facility layout problem (FLP) is the placement of facilities in a plant area where it is a significant component of the organization because it represents the organization's largest and most expensive assets (Figs. 2, 3). Theoretically, a graph is one approach to heuristic theories for solving the layout problem: when the objective is to maximize profit, the facility layout problem is to determine, in a given edge-weighted graph G, a maximum weight planar subgraph^15,16^.

### Literature review

The evaluation was a step in the hospital's redesign, and the facility has been described as a complex undertaking studied and analyzed by many researchers. Many researchers have studied and analyzed this process. Furthermore, it must incorporate the technical requirements demanded by the current and modern medical needs and consider the functional requirements in collaboration of a variety of units. The planner must consider issues such as healing capabilities, stressful workforce environments, the anxiety experienced by patients, and the sustainability of the facility^17^. Patients expect a given health facility to be easy to navigate, have a friendly and welcoming front or reception area, have soothing interiors such as cool colors, meet spiritual needs, and be able to view nature or access daylight while in the facility. It must also consider the needs of the working staff at heart by taking notice of such issues as break rooms, the distances they travel to serve their patients, their proximity to the patients they handle, and nature interior needs, which allow performing tasks at optimal levels, reflecting the quality of the patient. Care they give^18^.

^19^ Presented a new construction algorithm for a computer-aided plant layout. The layouts are created using ALDEP, whereas the adjacency-based heuristic and the maximum adjacency-based objective are used to evaluate them. The solution was built based on objectively measurable mathematical expressions. According to the findings, the layout generated by the adjacency-based heuristic has a higher layout score than that generated by the automated layout design program (ALDEP), and the adjacency-based heuristic can generate a shorter material handling distance than ALDEP.

Many types of research have been conducted on redesigning healthcare facilities to meet the required standards at a certain level. Building a new hospital allows aligning the hospital's layout with the intended logistical idea. This method is used to assess the flexibility and adaptability of a design for operations management to a specific instance. The case chosen is about a new Dutch hospital built on a new site after the merger of two hospitals. The new hospital introduces twenty-first century airport operations management concepts for designing an outpatient clinic. The twenty-first-century airport's concept aims to use the hospital building space by centralizing the waiting areas^20,21^.

In^22^, a redesigned model for clinical pharmacy in a university hospital in Colombia was described, which is the hospital unit tasked with making purchases, compounding, distributing various items, and storing purposes in a hospital. The model was described to contribute significantly to the increased interventions, which was estimated to be 70%. There was a 134% cost reduction, and most pharmacists' time was devoted to patient care rather than administrative activities. The activities of technicians and technologists were more focused on patient care. Approach for assessing hospital building design in terms of operations management to ensure that the design is fit for purpose aids in the efficient and effective operation of healthcare facility processes in the present and the future. An evaluation approach is a valuable tool for assessing both functionality and the ability of a building design's operational control to meet future advancements.

A variety of automated algorithms have been used to assist layout planners in developing alternate layouts. An automated layout design program (ALDEP) was created to improve the existing layout, whereas the computerized relative allocation of facilities technique (CRAFT) was created to improve the existing layout. Better results will be obtained by investigating hypotheses that combine the two algorithms rather than using them separately. The goal of this study was to use ALDEP to determine the best plan for Jaya Mandiri and improve it with CRAFT. The enhanced layout of the CRAFT was the best layout based on the cost of material handling, manufacturing lead time, and adjacency-based score^23^.

## Methodology

**Figure 4:**
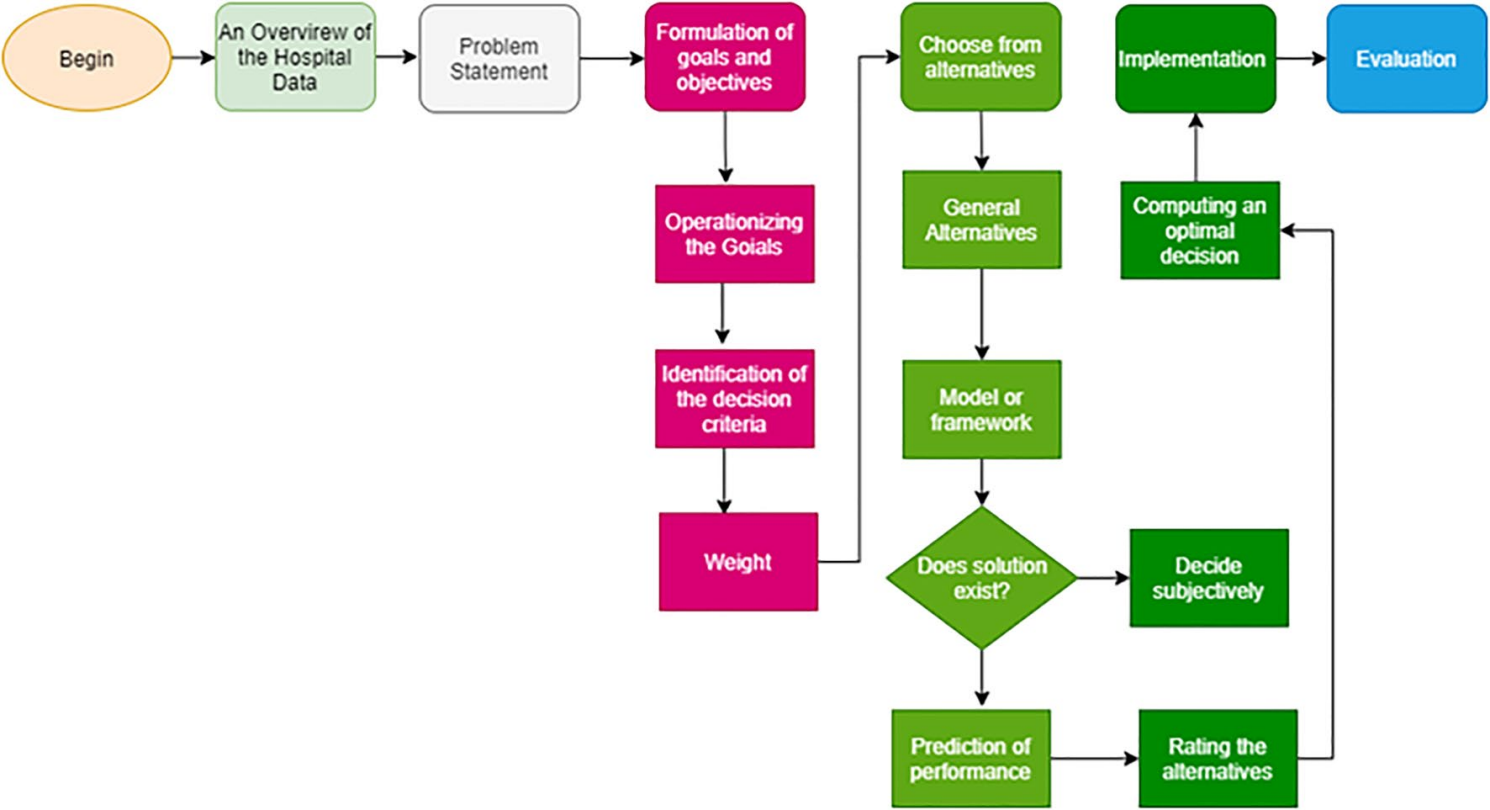
Evaluation model.

It is necessary to develop an algorithm that can be used in decision-making when developing algorithm models based on weighting standards. This study attempts to ensure that health facilities are evaluated in terms of their ability to provide quality standards. Most healthcare organizations face critical challenges when making decisions. It is difficult to obtain accurate undertakings in their operations for this purpose. In the current paper, the algorithms provide an overview of our approach to the weighting and ranking of hospitals for proper decisions, as shown in Fig. 4.

### Weighting algorithm

This study utilized the entropy weight method based on a literature review. It is frequently used to assess differentiation. The designed model incorporates various assessments to weigh the hospital according to the specifications of the entropy method. Algorithm 1^24^ is designed to ensure a proper analysis of hospital data and to consider particular conflicts occurring within the hospitals. Consequently, the multi-criteria decision-making (MCDM) issue is described as an ergonomic assessment in the workplace to monitor occupational disorders, and the criteria weight can be calculated^25^.

The weighting was calculated using MATLAB Version 2021b.

The evaluator is given a questionnaire containing the weighted criteria. Each criterion has a weight, and the answers are categorized into three choices (satisfied, can be satisfied, and not applicable) that can present the performance of each evaluated department."

**Table:** 

**Algorithm 1** Standards Weighting Using Entropy Method
1. Determine the importance of standards against another using pairwise comparisons, the relative importance of one criterion over another can be expressed" 1- Equal 3- Moderate 5- Strong 7- Very strong 9- Extreme by expertise commission 2. Build square matrix. With all the digits will be 1 in the diagonal of comparison matrix. First, the values of upper diagonal of the comparison matrix are filled with commission decision logic. To fill lower triangular matrix, upper diagonal is inverted. If ith is row element and jth is column element of a_ij_ matrix, lower diagonal is filled using a_ji_ = 1/a_ij_ formula 3. Normalization is done by dividing the criteria value by the total values of columns. (Normalizing Matrix P_ij_) using: ${\text{P}}_{{{\text{ij}}}} = \frac{x}{{\mathop \sum \nolimits_{i = 1}^{m} x_{ij} }}\quad (1)$ 4. Calculate $p_{{{\text{i}}j}}^{*} = P_{1 \cdot j} \ln P_{ij} \quad (2)$ 5. Sum the column totals $p_{ij}^{*}$ matrix 6. Calculate ${\text{E}}_{{{\text{i}}\dot{\text{{j}}}}} = - 1{/}\ln (m)*p^{*} \quad (3)$ 7. Calculate $\mathop \sum \limits_{{ - {\text{j}}}}^{{\text{n}}} 1 - {\text{E}}_{{{\text{ij}}}} \quad (4)$ 8. The criteria weight can be calculated by: $w = \frac{{1 - E_{ij} }}{{\mathop \sum \nolimits_{j = 1}^{n} (1 - E_{ij} )}}\quad (5)$

### Layout score

The weights of the edges must be defined to calculate the layout score of each department (service) using graph heuristic theory. They represent the benefits of having two adjacent spaces. The weights between spaces are classified according to the AEIOUX rating system.

### Activity relationship chart

Represents M (M − 1)/2 symmetric qualitative relationships, where M is the number of spaces in the department layout. The degree of this relationship can be described using the AEIOUX rating^26,27^. Table 1 depicts the proposed REL chart of each department using the AEIOUX rating system according to international standards, as shown in Algorithm 2.

**Table 1:** AEIOUX rating system, definitions and values.

Symbol	Value	Requirements for spaces to be close
A	4	Absolutely necessary
E	3	Especially important
I	2	Important closeness
O	1	Ordinary closeness
U	0	Unimportant
x	− 1, − 2, − 3	Undesirable

#### Adjacent matrix

The vertices represent the spaces within the department layout, and the edges represent the "adjacencies" The proposed adjacency coefficients are as follows:

Fully adjacent (take value 1): Two spaces are (fully) adjacent in a layout if they only share one wall or two spaces face each other directly.

Partially adjacent (take values 0.5 of 0.75): Two spaces are partially adjacent in a layout if these spaces are together in the same area. The space closer to the selected space was 0.75, and the farthest space had a value of 0.5.

Non-adjacent (take value 0): If the spaces do not share any point or cannot be seen together in the same area (a door or curtain separates them)^19,28,29^.

The layout plan for each department was drawn using AutoCAD program version 2019.

Let a_ij_ *ϵ* [0, *α*, 1] be the adjacency coefficient between activities i and j.

6 $$a_{ij} = \left\{ {\begin{array}{*{20}l} 1 \hfill & {if\;i\;and\;j\;are\;fully\;adjacent.} \hfill \\ \alpha \hfill & {if\;i\;and\;j\;are\;partially\;adjacent.} \hfill \\ 0 \hfill & {if\;i\;and\;j\;are\;not\;adjacent.} \hfill \\ \end{array} } \right.$$

where a_ij_ *∈* [0, 1]: adjacency coefficient between activities i and j. V (r)_ij_ or W (r)_ij_ is a weighting factor described in the AEIOUX rating system section.

**Table:** 

**Algorithm 2** Calculation of Layout Plan Score using graph Heuristic Method
1. Generate a Relationship matrix for each department according to AEIOUX Rate 2. Generate an adjacency matrix from the existing layout plan using $$a_{ij} = \left\{ {\begin{array}{*{20}l} 1 \hfill & {if\;i\;and\;j\;are\;fully\;adjacent.} \hfill \\ \alpha \hfill & {if\;i\;and\;j\;are\;partially\;adjacent.} \hfill \\ 0 \hfill & {if\;i\;and\;j\;are\;not\;adjacent.} \hfill \\ \end{array} } \right.\quad (6)$$ 3. Calculate Layout score based on adjacency Using ${\text{LS}}^{{\text{a}}} { = }\mathop \sum \limits_{{\text{i = 1}}}^{{\text{M - 1}}} \mathop \sum \limits_{{\text{j = j + 1}}}^{{\text{M}}} {\text{V(r}}_{{{\text{ij}}}} {)}{\text{.a}}_{{{\text{ij}}}} \quad (7)$

## Results and discussion

The proposed method was applied to hospitals in Egypt, and the selected hospital was a public governmental hospital. It includes several departments and services as public hospitals. This method is applied to evaluate hospitals according to international standards^30^ and measure the layout plan score to help us determine if redesign can be done for this department to meet international standards.

### The selected hospital description

**Figure 5:**
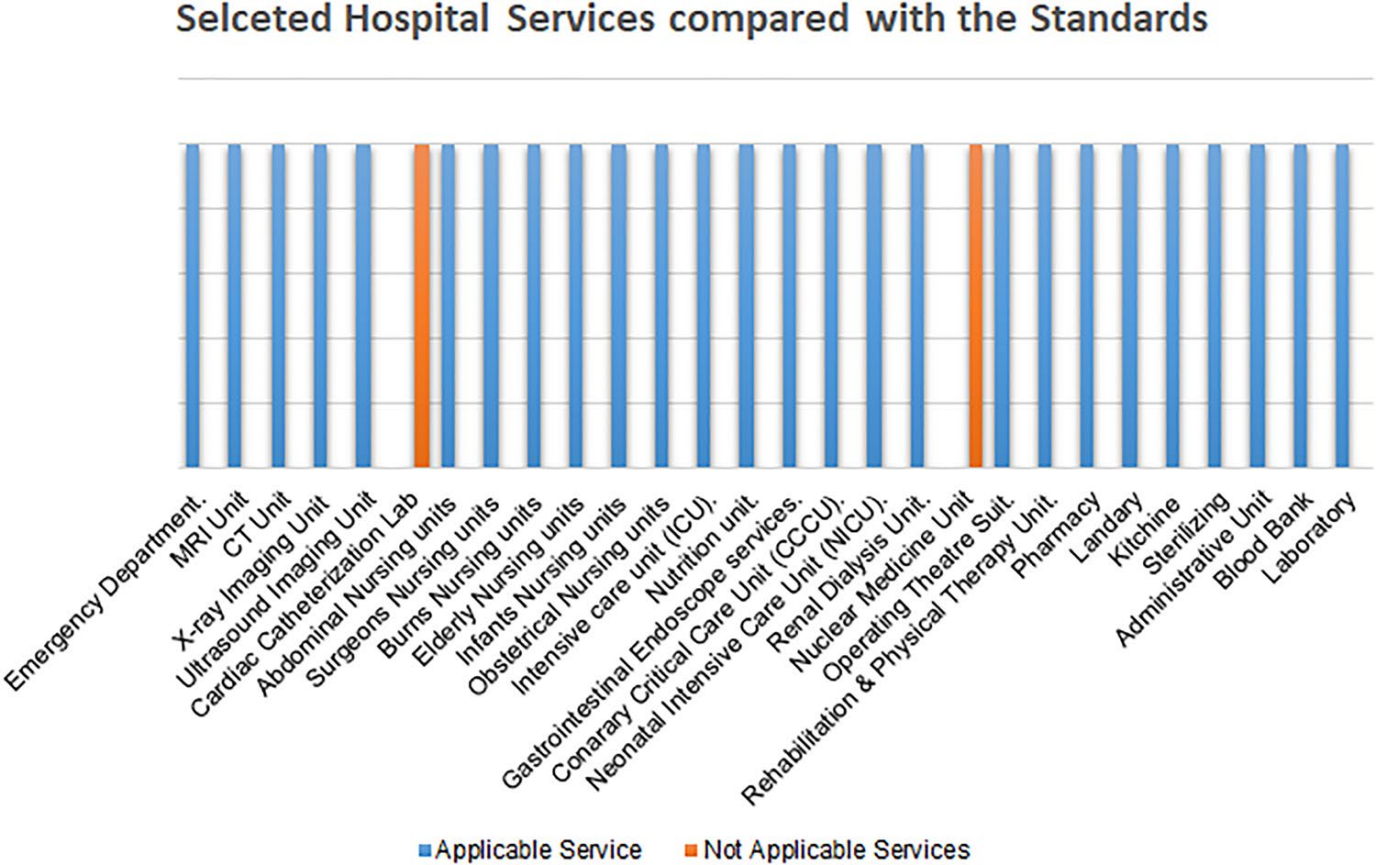
The services in the selected hospital versus the standard services for general hospitals.

The selected hospital is a public hospital that must contain the following departments according to the collected international standards, as shown in Fig. 5.

**Figure 6:**
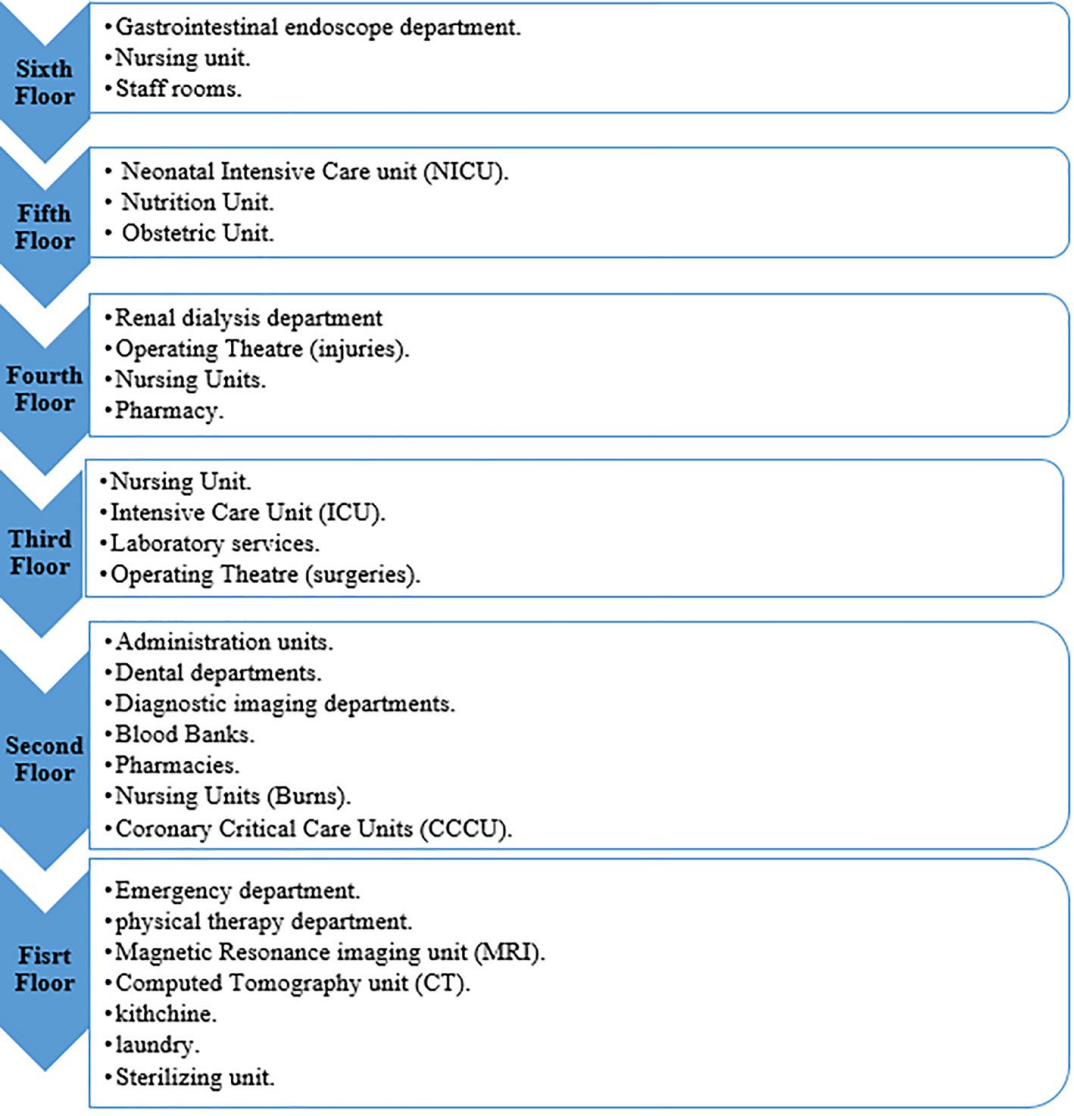
The distribution of departments and services in the selected hospital.

Assessment of the new model was performed by evaluating each hospital department. The selected hospital consisted of two buildings. The first is the main building, consisting of six floors, as shown in Fig. 6.

### The selected hospital evaluation

#### Emergency department

**Figure 7:**
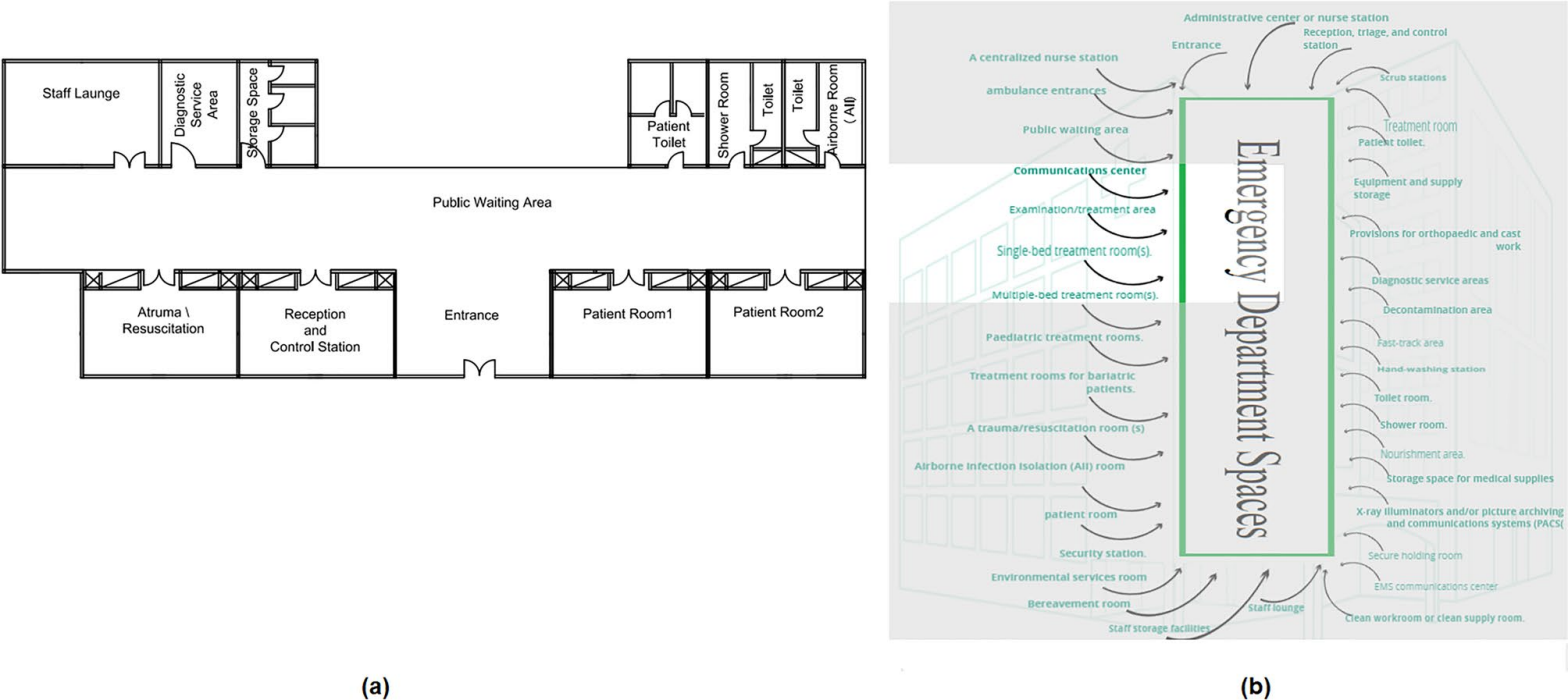
(**a**) Emergency department layout plan in the selected hospital (**b**) emergency department areas standards.

As demonstrated in Fig. 7a, the spaces that are satisfied and the spaces that are not applicable in the ED are observed by comparing the existing layout with the standards area, as shown in Fig. 7b and calculating layout score of existing ED.

#### Operating theatre suites

**Figure 8:**
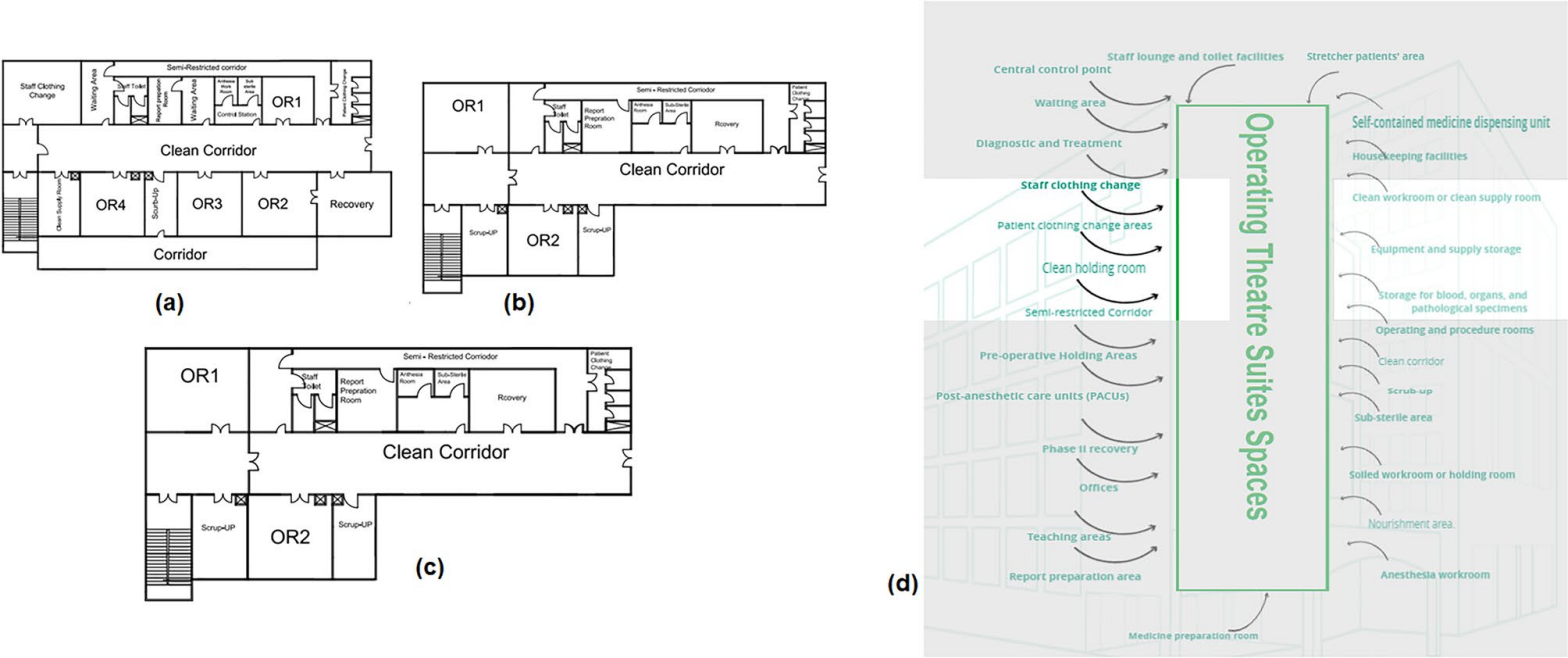
Operating theatre layout plan in the selected hospital (**a**) injuries, (**b**) surgeries, **c** obstetrical, (**d**) operating theatre areas standards.

As shown in Fig. 8a–c, the spaces that are satisfied and the spaces that are not applicable in the Operating theatre (Injuries), (Surgeries), and (Obstetrical) are observed by comparing the existing layout with the standards area, as shown in Fig. 8d and calculating the layout score of existing Operating Theatre Layout suites.

#### Nursing units

**Figure 9:**
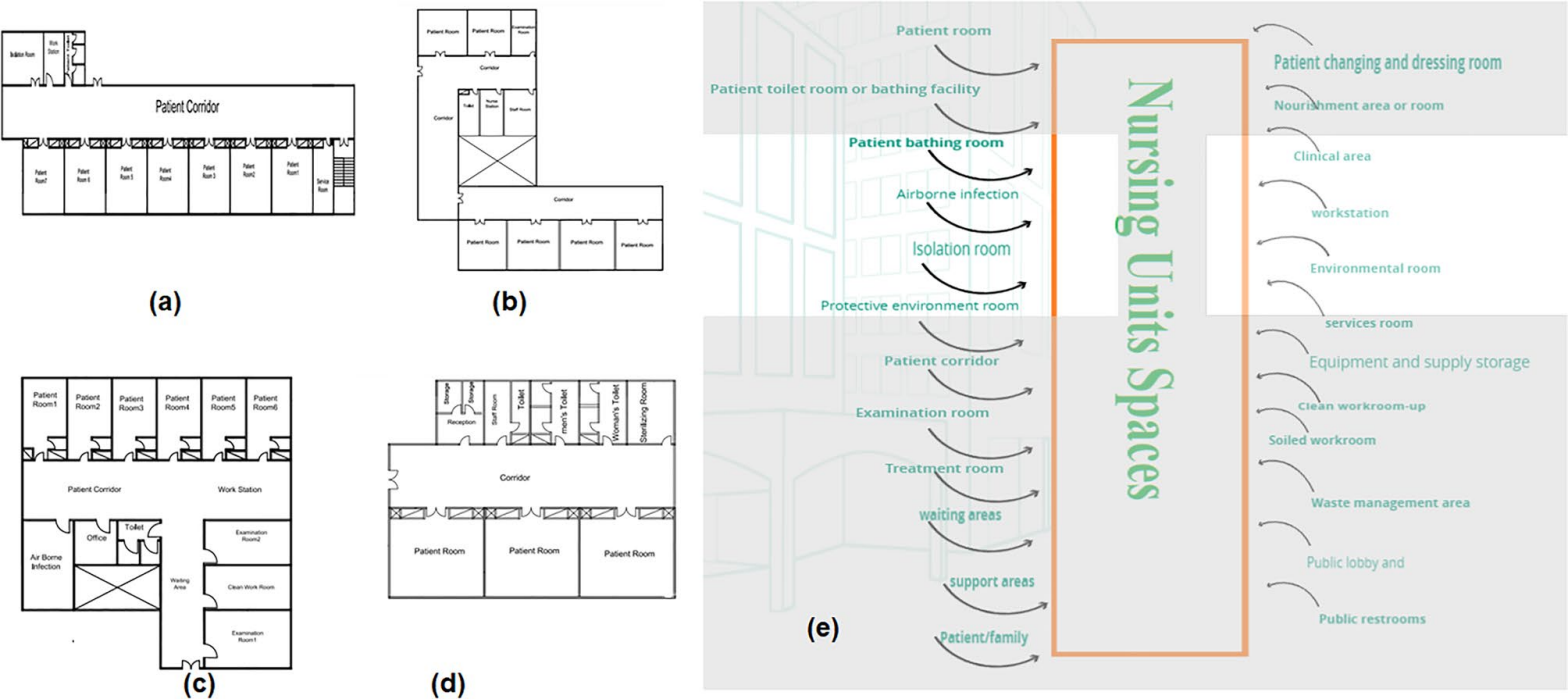
Nursing unit layout in the selected hospital (**a**) abdominal, (**b**) obstetrical, (**c**) burns, (**d**) Elder's, (**e**) standards nursing unit areas.

The spaces that are satisfied and the spaces that are not applicable in the nursing units (Abdominal), (Obstetrical), (Burns), and (Elder's)) observed from comparing the existing layout with the standards area as demonstrated in Fig. 9a–d when calculating the layout score of existing Nursing Units.

#### Laboratory services

**Figure 10:**
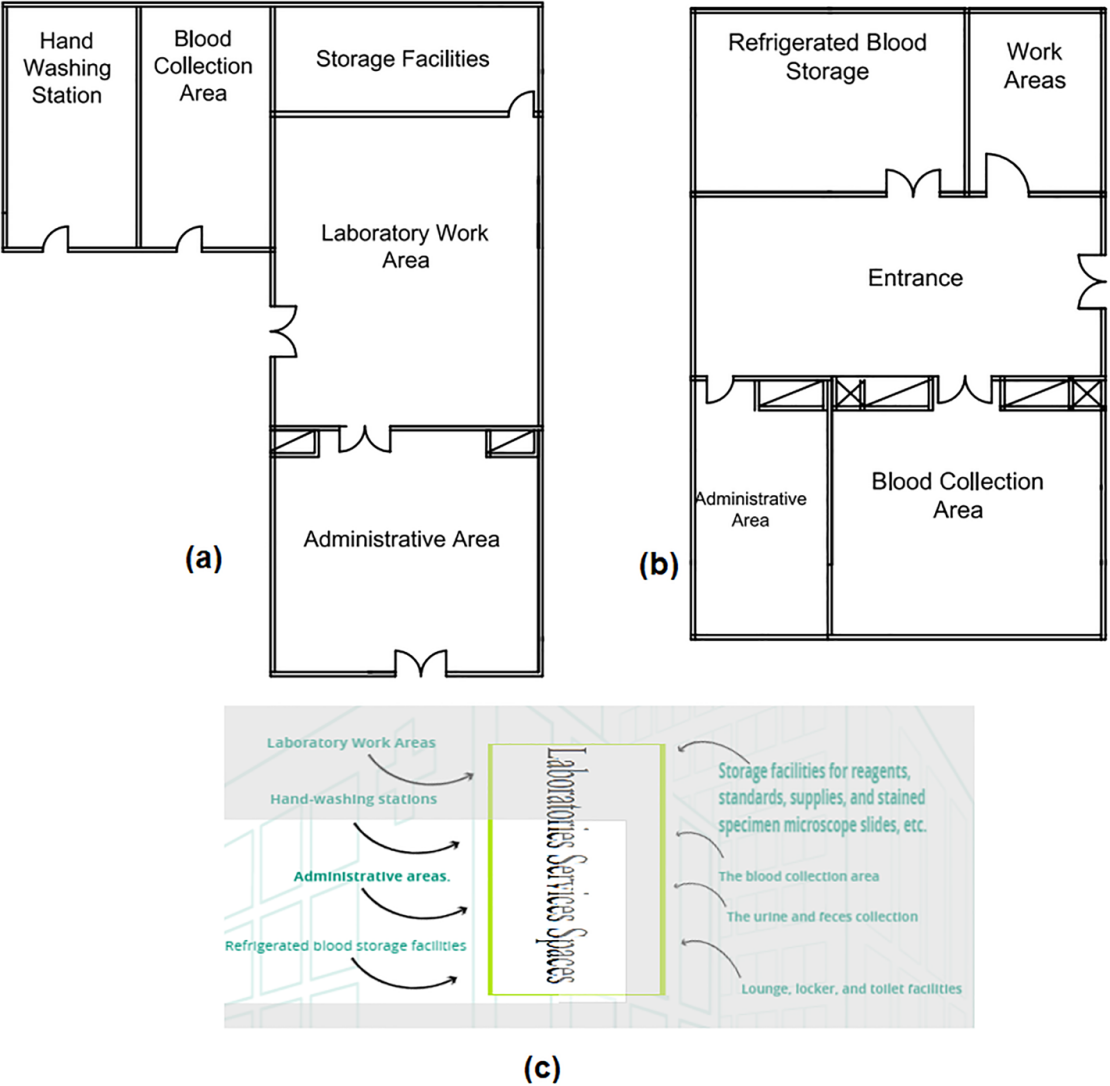
Laboratoesis service layout plan in the selected hospital (**a**) main lab, **b** blood bank, **c** standards laboratoesis service areas.

The spaces that are satisfied and the spaces that are not applicable in the Laboratoesis Service (Main lab) (Blood Bank) are observed by comparing the existing layout with the standards area, as demonstrated in Figs. 9a, b, and 10c, which can be used to calculate the layout score of the existing Laboratoesis Services.

#### Coronary critical care unit (CCCU)

**Figure 11:**
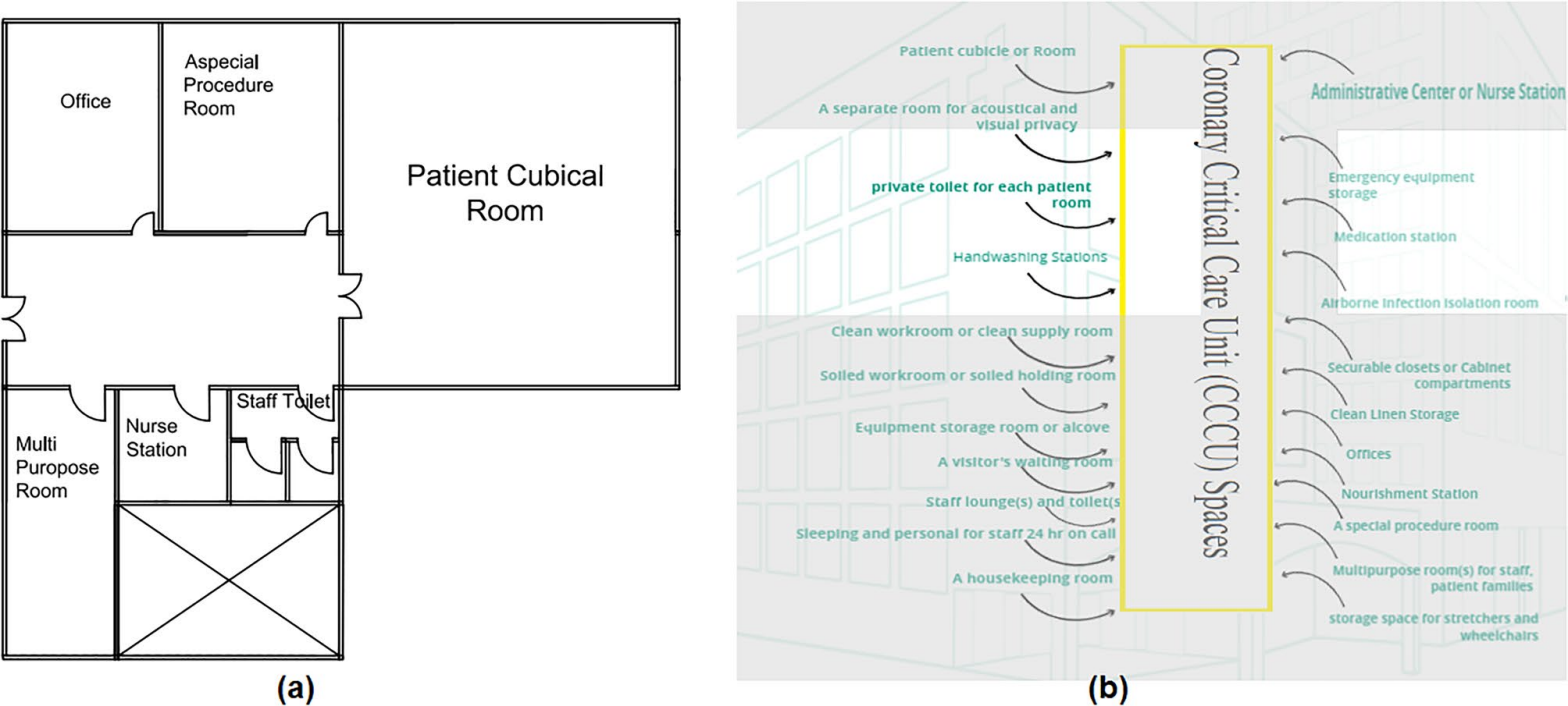
(**a**) Coronary critical care unit (CCCU) layout plan in the selected hospital, (**b**) standards coronary critical care unit (CCCU) areas.

The spaces that are satisfied and the spaces that are not applicable in the CCCU are observed by comparing the existing layout with the standards area, as shown in Figs. 10b and 11a, which can be used to calculate the layout score of the existing CCCU**.**

#### Intensive care unit (ICU)

**Figure 12:**
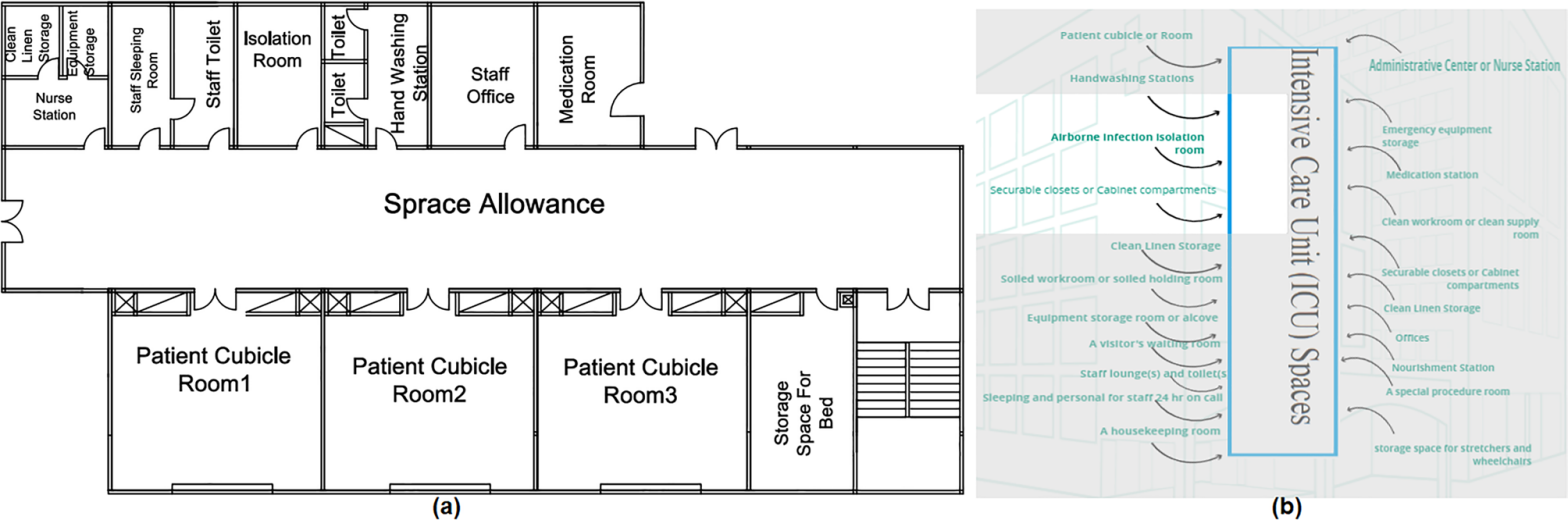
(**a**) Intensive care unit layout plan in the selected hospital, (**b**) standards intensive care unit (ICU) areas.

The spaces that are satisfied and the spaces that are not applicable in the (ICU are observed by comparing the existing layout with the standards area as shown in Fig. 12a, b, which can be used to calculate the layout score of the existing ICU.

#### Nurseries unit

**Figure 13:**
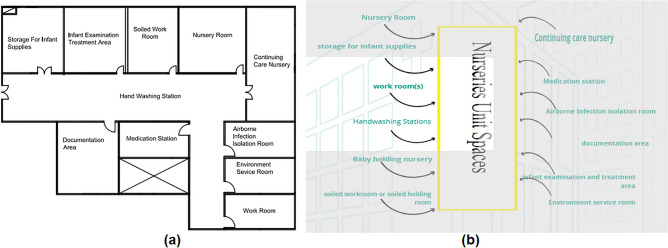
(**a**) Nurseries unit layout plan in the selected hospital, (**b**) standards nurseries unit areas.

The spaces that are satisfied and the spaces that are not applicable in the Nurseries unit are observed by comparing the existing layout with the standards area, as shown in Fig. 13a, b, which can be used to calculate the layout score of the nurseries unit.

#### Neonatal intensive care unit (NICU)

**Figure 14:**
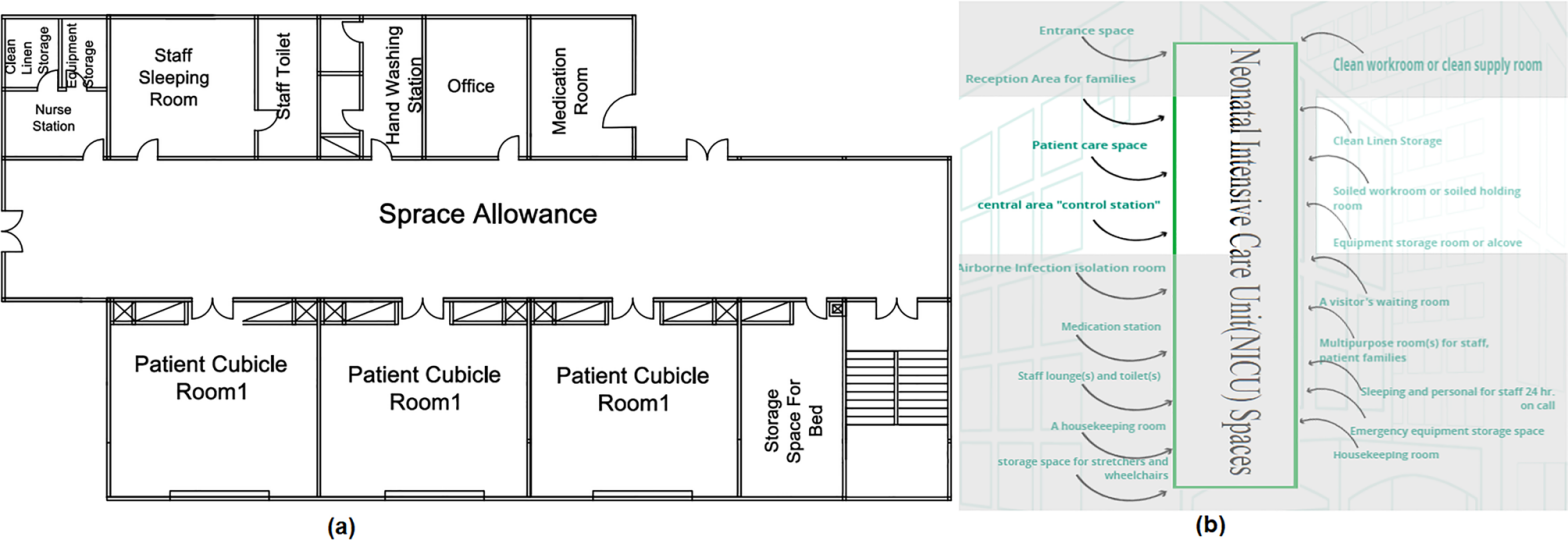
(**a**) Neonatal intensive care unit (NICU) layout plan in the selected hospital, (**b**) standards neonatal intensive care unit (NICU) areas.

The spaces that are satisfied and the spaces that are not applicable in the NICU are observed by comparing the existing layout with the standards area, as shown in Fig. 14a, b, which can calculate the layout score in the existing NICU.

#### Gastrointestinal endoscopy department

**Figure 15:**
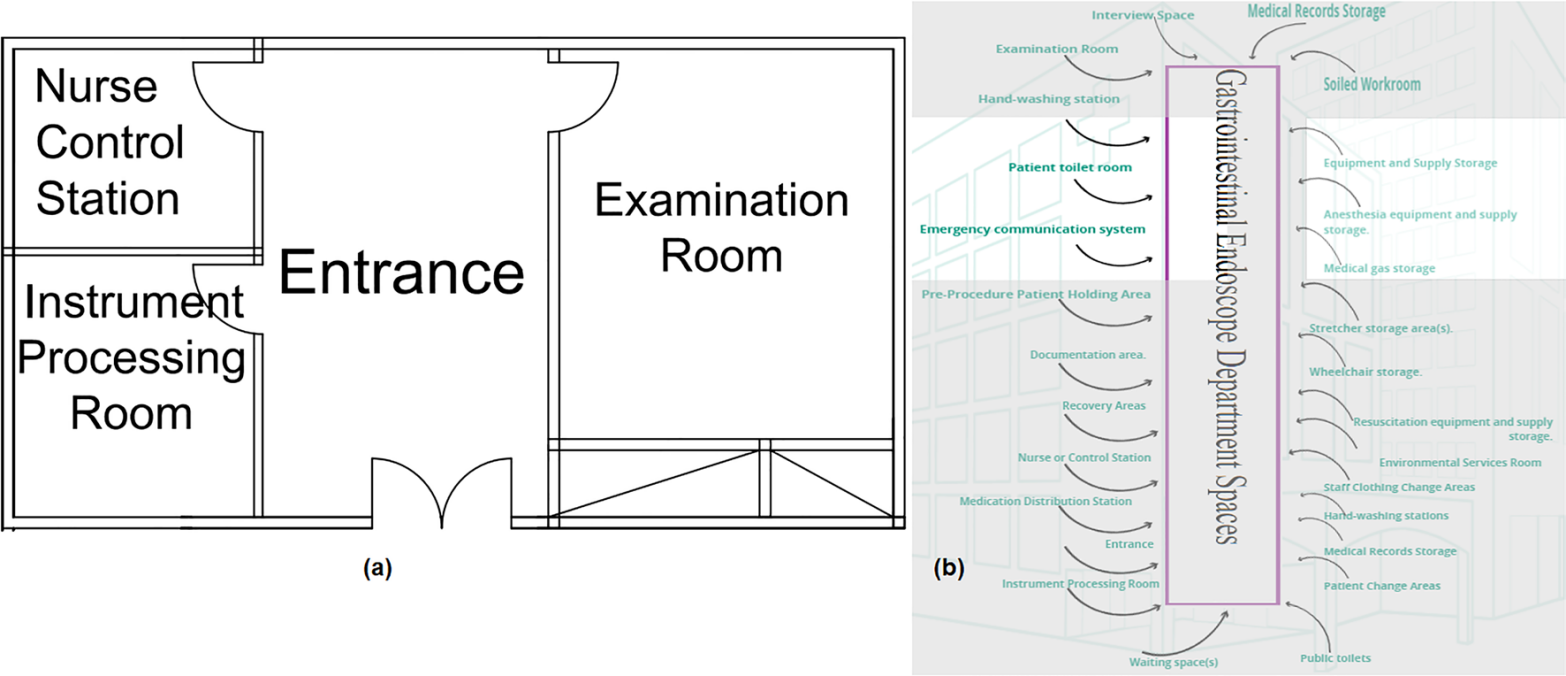
(**a**) Gastrointestinal endoscope department layout plan in the selected hospital, (**b**) standards gastrointestinal endoscope department areas.

The spaces that are satisfied and the spaces that are not applicable in the Gastrointestinal Endoscope Department are observed by comparing the existing layout with the standards area, as shown in Fig. 15a, b, which can be used to calculate the layout score of the Gastrointestinal Endoscope Department.

#### Renal dialysis department

**Figure 16:**
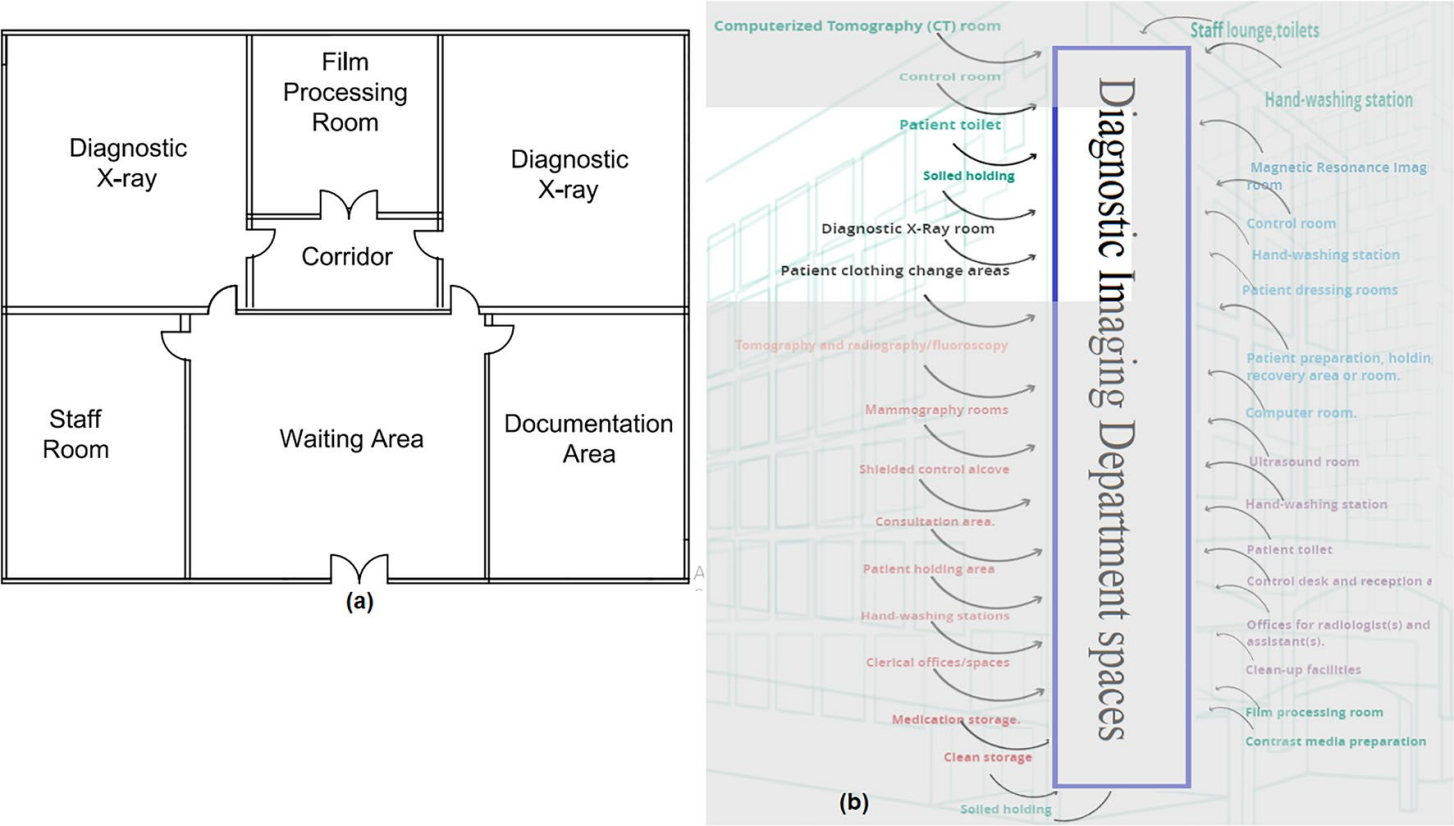
(**a**) Renal dialysis department layout plan in the selected hospital, (**b**) standards renal dialysis areas.

The spaces that are satisfied and the spaces that are not applicable in the Renal Dialysis Department are observed by comparing the existing layout with the standards area as shown in Fig. 16a, b, which can calculate the layout score of the Renal Dialysis Department.

#### Diagnostic imaging department

**Figure 17:**
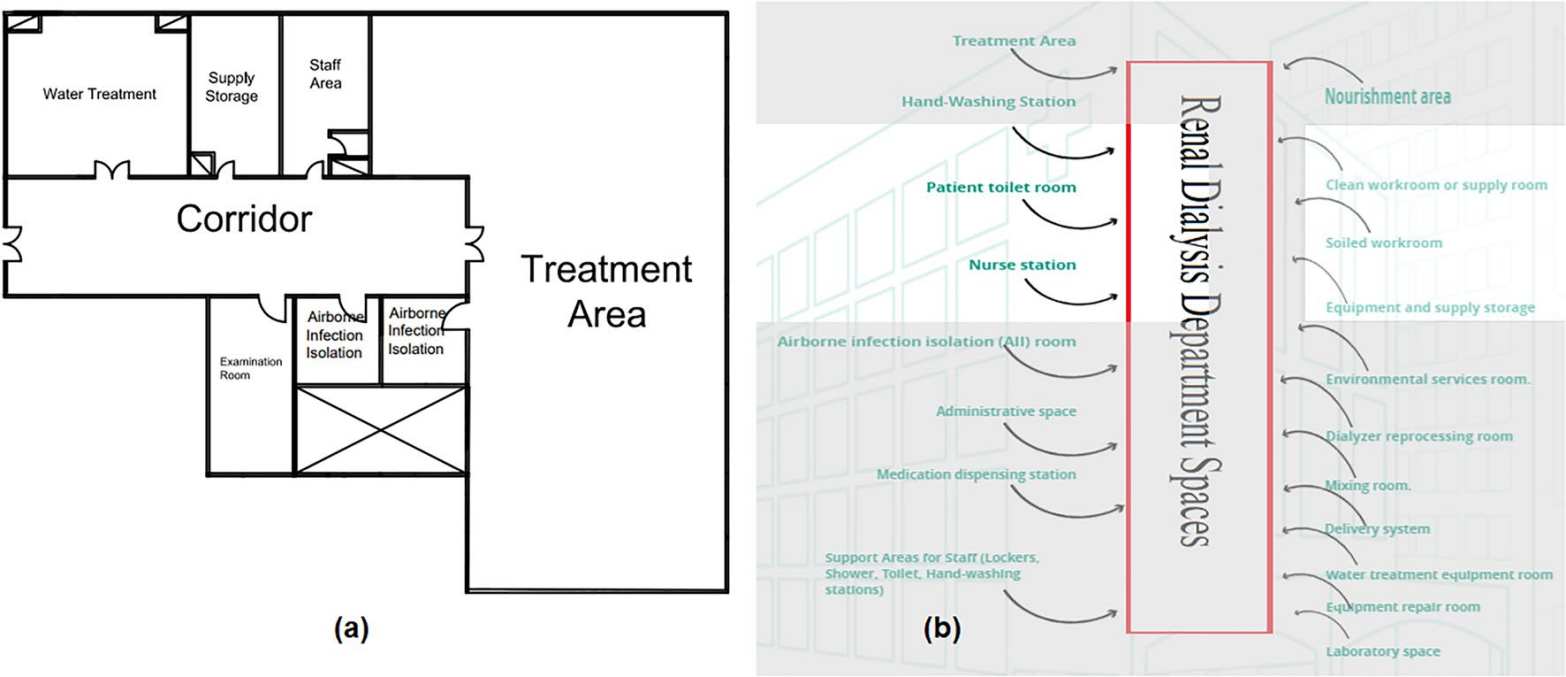
(**a**) Diagnostic imaging department layout plan in the selected hospital, (**b**) diagnostic imaging department areas.

The spaces that are satisfied and those that are not applicable in the Diagnostic Imaging Department are observed by comparing the existing layout with the standards area, as shown in Fig. 17a, b, which can be used to calculate the layout score of the Diagnostic Imaging Department.

#### Rehabilitation physical therapy department

**Figure 18:**
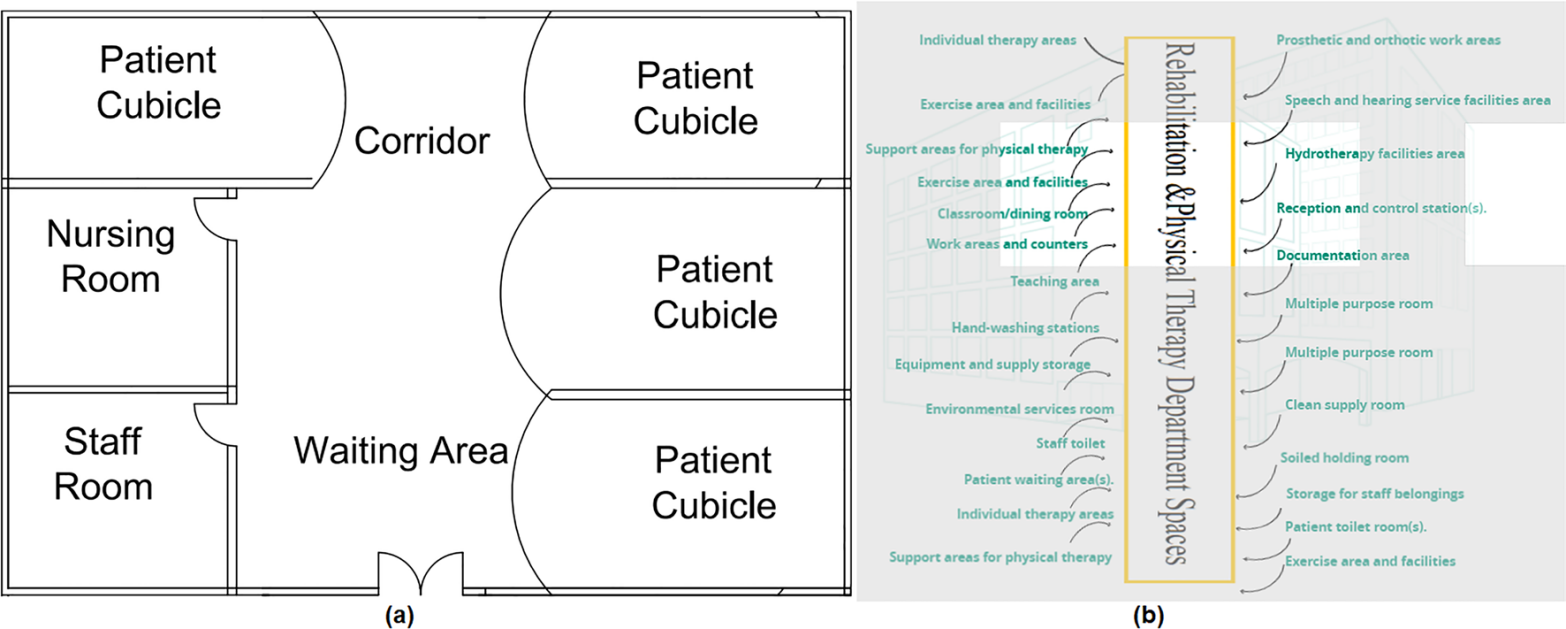
(**a**) Rehabilitation & physical therapy department layout plan in the selected hospital, (**b**) standards rehabilitation & physical therapy department areas.

The spaces that are satisfied and those not applicable in the Department of Rehabilitation and Physical Therapy are observed by comparing the existing layout with the standards area as shown in Fig. 18a, b, which can be used to calculate the layout score of the Rehabilitation and Physical Therapy department.

#### Evaluation results

The international standards for the design of healthcare facilities have been compiled and categorized into criteria. Each criterion in the hospital department is weighted using entropy, with the sum of all of the department criteria equaling one. The evaluation of each department's criteria takes the form of a questionnaire, to which the expert evaluator can respond with one of three options (satisfied, can be satisfied, not applicable). Based on these responses, we can calculate the satisfaction percentage of hospital department criteria and other options.

**Figure 19:**
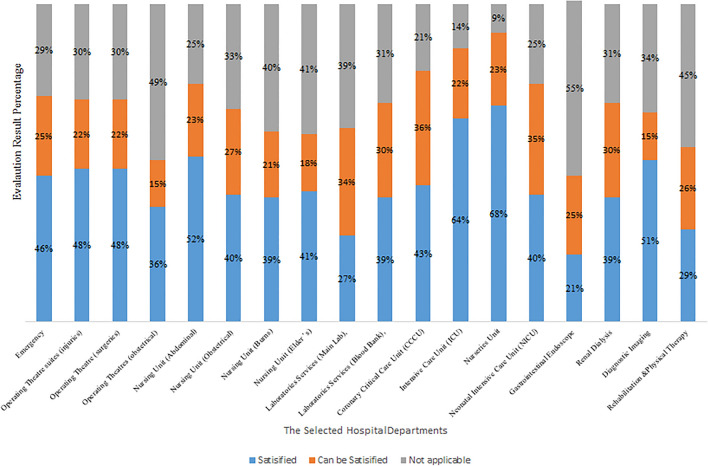
The evaluation results of the selected hospital departments.

Figure 19 depicts the percentage of stratified weighted criteria that can be satisfied and those that are not applicable after applying the evaluation methodology to selected hospital departments.

**Figure 20:**
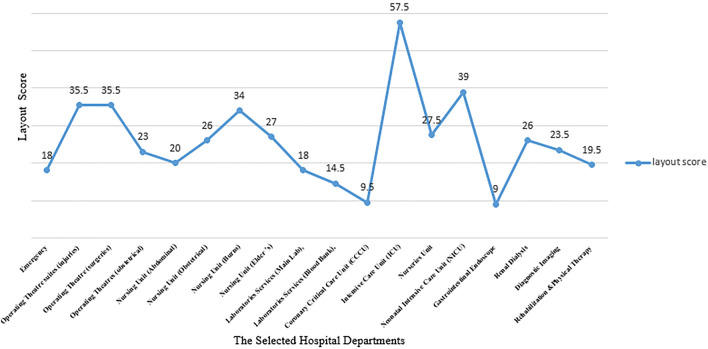
The selected hospital departments layout score.

The layout score is calculated using Algorithm 2, in which we build a relationship matrix and build an adjacency matrix for each department, and then using Eq. (7), the layout score of each department is calculated and graphically presented, as shown in Fig. 20. For example, the calculation of emergency departments as:

First Step: Generate an adjacency matrix from the existing ED layout plan (Fig. 7a) by considering adjacency coefficient definitions in the Methodology Section:

Second step: Generate a relationship matrix from the existing ED layout plan (Fig. 7a) by considering spaces relationship definitions in the Methodology Section:

Third step: Calculate the layout score by using Eq. (7) at Algorithm 2 (layout score based on adjacency) and replace the letters of the AEIOUX rating system by their values, as it was mentioned in (Table 2). The resulted equation will be as the following:

$$\begin{aligned} & {\text{Layout Score}} = \\ & \quad {\text{A*0}}{\text{.5 + X*1 + X*1 + X*1 + X*0}}{\text{.5 + E*0}}{\text{.5 + A*0}}{\text{.5 + U*0}}{\text{.5 + E*0}}{\text{.5 + I*0}}{\text{.5 + I*0}}{.5} \\ & \quad \quad {\text{ + I*0}}{\text{.5 + I*0}}{\text{.5 + A*1 + I*0}}{\text{.5A*1 + U*0}}{\text{.5 + A*0}}{\text{.5 + U*1 = 18}}{.} \\ \end{aligned}$$

**Table 2:** Emergency department spaces in the selected hospital.

Spaces	Satisfied	Not applicable
Entrance		
Emergency department ambulance entrances		
Reception, triage, and control station		
Treatment room		
Patient toilet		
Equipment and supply storage		
Public waiting area		
Communications center		
Examination/treatment room or area		
Single-bed treatment room(s)		
Multiple-bed treatment room(s)		
Pediatric treatment rooms		
Treatment rooms for bariatric patients		
A trauma/resuscitation room(s)		
Provisions for orthopedic and cast work		
Diagnostic service areas		
Decontamination area		
Fast-track area		
Airborne infection isolation (AII) room		
patient room		
A centralized nurse station		
Hand-washing station		
Toilet room		
Shower room		
Nourishment area		
Storage space for medical supplies shall be provided under staff control		
X-ray illuminators and/or picture archiving and communications systems (PACS)		
Secure holding room		
Administrative center or nurse station		
Security station		
EMS communications center		
Scrub stations		
Provisions for disposal of solid and liquid waste		
Clean workroom or clean supply room		
Soiled workroom or soiled holding room		
Equipment and supply storage		
Environmental services room		
Staff lounge		
Staff storage facilities		
Bereavement room		

And thus, the layout score can be calculated for every department in the selected hospital (Tables 3, 4).

**Table 3:** ED REL matrix in selected hospital.

	Area	1	2	3	4	5	6	7	8	9	10	11
1	Entrance		A	U	A	A	O	A	O	O	O	O
2	Equipment and supply storage			A	O	O	O	O	O	O	O	O
3	Staff lounge				O	E	O	O	O	O	O	O
4	Public waiting area					A	U	E	I	I	I	I
5	Diagnostic service areas						X	I	E	I	E	I
6	Patient toilet							A	I	O	O	O
7	Shower room								A	O	E	I
8	Airborne infection isolation (AII) room									U	A	E
9	Multiple-bed treatment room(s)										U	A
10	Reception, triage, and control station											U
11	A trauma/resuscitation room(s)

**Table 4:** ED adjacency matrix in selected hospital.

	Area	1	2	3	4	5	6	7	8	9	10	11
1	Entrance		0	0	0.5	0	0	0	0	1	1	0
2	Equipment and supply storage			0	0	1	0	0	0	0	0	0
3	Staff lounge				0.5	0.5	0	0	0	0	0	0
4	Public waiting area					0.5	0.5	0.5	0.5	0.5	0.5	0.5
5	Diagnostic service areas						0	0	0	0	0	0
6	Patient toilet							1	0.5	0	0	0
7	Shower room								1	0	0	0
8	Airborne infection isolation (AII) room									0	0	0
9	Multiple-bed treatment room(s)										0.5	0.5
10	Reception, triage, and control station											1
11	A trauma/resuscitation room(s)											

The layout score for each department in the selected hospital is demonstrated in Fig. 20.

After applying the study methodology at the selected hospital, the analysis of evaluation results revealed that the intensive care unit is the most satisfied department according to international standards (ICU). While the department with the lowest satisfaction according to international standards is the Gastrointestinal Endoscope Department. Most departments that form the layout score are the intensive care unit (ICU), and the lowest department from the layout score is the Gastroendoscope Department, as shown in Fig. 19.

The average of the satisfied standards was 43%, the standards that could be satisfied were 24%, the standards that were not applicable were 34%, and the average layout score was 25. The design evaluation that helps to improve the existing layout using reallocation or combination of more than one area to meet the design standards is integrated and includes the required functionalities.

## Conclusion

The analysis of the results revealed that there are no nuclear medicine departments or cardiac catheterization laboratories in the selected hospital, although they must be included in a public hospital. The overall selected hospital lacks environmental room(s) and ventilation standards. More than operating theatre suites are rearranged to meet international standards. This proposed model is a step in the redesign process and has many benefits, such as reducing the percentage of errors that occur due to human design as well as time and effort savings. The result of this study helps in developing the selected hospital to provide more services, increase the capacity of patients, reduce the time for providing medical services, and create an appropriate and comfortable environment for medical staff and patients. This model does not require definitive knowledge of international design standards and aids in the redesign of a hospital as a developmental process. In future work, our model will be expanded into intelligent hospitals, study the effect of the restrictions on the operation of the proposed algorithm, and generate a software programme to handle all problems of hospital layout design, with more considerations such as area, distance, flow, cost, and time, and study the reallocation of spaces to improve the layout of each department.

## Data Availability

The data supporting this study's findings are available from the corresponding author upon reasonable request.
